# Co-design of a Mobile App for Engaging Breast Cancer Patients in Reporting Health Experiences: Qualitative Case Study

**DOI:** 10.2196/45968

**Published:** 2023-11-27

**Authors:** Carla Taramasco, Carla Rimassa, René Noël, María Loreto Bravo Storm, César Sánchez

**Affiliations:** 1 Instituto de Tecnologías para la Innovación en Salud y Bienestar, Facultad de Ingeniería Universidad Andrés Bello Viña del Mar Chile; 2 Centro para la Prevención y el Control del Cáncer Santiago Chile; 3 Facultad de Medicina, Escuela de Fonoaudiología, Campus San Felipe Universidad de Valparaíso San Felipe Chile; 4 Escuela de Ingeniería Informática, Facultad de Ingeniería Universidad de Valparaíso Valparaíso Chile; 5 Departamento de Hematología y Oncología, Escuela de Medicina Pontificia Universidad Católica de Chile Santiago Chile

**Keywords:** cancer, registration systems, patient-reported outcome measures, patient-reported experience measures, software analysis and design, cancer patient report, adverse event reporting, quality of life, eHealth, mHealth, mobile health

## Abstract

**Background:**

The World Health Organization recommends incorporating patient-reported experience measures and patient-reported outcome measures to ensure care processes. New technologies, such as mobile apps, could help report and monitor patients’ adverse effects and doubts during treatment. However, engaging patients in the daily use of mobile apps is a challenge that must be addressed in accordance with the needs of people.

**Objective:**

We present a qualitative case study documenting the process of identifying the information needs of breast cancer patients and health care professionals during the treatment process in a Chilean cancer institution. The study aims to identify patients’ information requirements for integration into a mobile app that accompanies patients throughout their treatment while also providing features for reporting adverse symptoms.

**Methods:**

We conducted focus groups with breast cancer patients who were undergoing chemotherapy (n=3) or who completed chemotherapy between 3 months and 1 year (n=1). We also surveyed health care professionals (n=9) who were involved in patient care and who belonged to the oncology committee of the cancer center where the study took place. Content analysis was applied to the responses to categorize the information needs and the means to satisfy them. A user interface was designed according to the findings of the focus groups and was assessed by 3 trained information system and user interaction design experts from 2 countries, using heuristic evaluation guidelines for mobile apps.

**Results:**

Patients’ information needs were classified into 4 areas: an overview of the disease, information on treatment and day-to-day affairs, assistance on the normality and abnormality of symptoms during treatment, and symptoms relevant to report. Health care professionals required patients to be provided with information on the administrative and financial process. We noted that the active involvement of the following 4 main actors is required to satisfy the information needs: patients, caregivers, social network moderators, and health professionals. Seven usability guidelines were extracted from the heuristic evaluation recommendations.

**Conclusions:**

A mobile app that seeks to accompany breast cancer patients to report symptoms requires the involvement of multiple participants to handle the reports and day-to-day information needs. User interfaces must be designed with consideration of the patient’s social conventions and the emotional load of the disease information.

## Introduction

### Background

Cancer is a disease characterized by the accelerated multiplication of abnormal cells that are able to spread to different organs (metastasis), which is the main cause of death worldwide, with almost 10 million deaths in 2020, and breast, lung, colorectal, prostate, dermal, and gastric cancers are the most common [1]. The situation in Latin America is worrying because survival after 5 years of diagnosis is lower than that in Organization for Economic Co-operation and Development (OECD) countries [2]. In Chile, as in other countries of the world, there has been an increase in morbidity and mortality from acute and chronic noncommunicable diseases (NCDs). Specifically, at the national level between 2009 and 2019, NCDs were the main cause of death, with cancer ranked first and cardiovascular diseases ranked second in 2019, and Chile has been placed second in South America [3]. In addition, although the disease can affect people throughout their life cycle, data from the beginning of the second millennium have shown that the number of new cases among both sexes increases with advancing age [4].

In Chile in recent years, certain milestones have been achieved that are together aimed at improving the detection, care, and monitoring of people with cancer, among which are the National Cancer Plan of 2018 [4], the National Cancer Law (Law 21 258) [5], and the National Cancer Registry (NCR) [6]. In the National Cancer Plan, 5 strategic areas are proposed, 3 of which are considered transversal and fundamental, including the strengthening of registration, information, and surveillance systems [7]. The Cancer Law mentions mandatory notification of the disease [5], and the NCR provides health authorities with a national information system that continuously and systematically collects, stores, processes, and analyzes data on all cases and types of cancers that occur in the country, which includes public and private health patients, and more than 20 establishments, with over 5000 cases of cancer recorded [2].

The NCR is a technological tool that helps monitor cancer trends over time, guides the planning and evaluation of cancer control programs, shows cancer patterns in different populations, and identifies high-risk groups, enabling decisions to be made with specific needs in mind, and information from the NCR contributes to prioritizing resource allocation and promoting research activities in specific areas [3]. However, the World Health Organization now recommends that to guarantee health care processes, it is important to incorporate patient-reported experience measures (PREMs) and patient-reported outcome measures (PROMs) [8]. This health entity mentions that quality assurance and improvement are important components of the development and sustainability of services that must consider cultural characteristics. Thus, outcome measures reported by patients and health professionals, and measures of experience provide valuable data on the person’s centrality and effectiveness regarding the services provided, presenting information on a person’s self-perception of their health, which may include quality of life, functioning, and self-efficacy, or revealing a person’s perception of their experience of a service of health or social care, which may include experience in terms of access, waiting times, and the possibility of participating in shared decision-making [8]. Therefore, it is necessary to incorporate information directly from patients into the NCR.

Mobile apps for collecting and managing adverse symptoms have been designed [9,10], and experimental evidence confirms their positive effects among breast cancer patients, leading to significantly less symptom prevalence and symptom burden [11]. However, engaging patients in the daily use of apps for reporting health experiences is challenging [12]. One strategy to introduce a tool for reporting adverse symptoms into the daily life of breast cancer patients and simultaneously influencing their quality of life is to leverage positive experiences with accompanying applications [13,14]. While there is evidence that functionalities, such as discussion and learning forums, are highly used by patients, adherence to use depends on various factors, requiring a tailored design [15].

In this study, we aimed to identify the information needs of patients and health care professionals in order to integrate them into a mobile app that accompanies patients throughout their treatment and, at the same time, offers functions for reporting adverse symptoms. The following subsections present evidence on the benefits of reporting adverse symptoms through PREM and PROM surveys, and existing technological tools to support this process.

### Use of PREMs and PROMs in Cancer

The clinical follow-up of cancer patients is an interdisciplinary activity that aims to control side effects and detect early possible relapse, which varies depending on the type of cancer and characteristics of the person [4]. In Chile, follow-up is part of the treatment of cancer patients and is carried out through secondary and tertiary care [16] to monitor possible complications of the disease (metastasis, thrombosis, dysphagia, etc) and treatment (myopathies, neuropathies, etc) [17]. However, such information, which focuses on estimating the incidence and type of cancer, is not collected in the NCR or used in nationwide statistics. The NCR collects information from 4 population-based cancer registries in the country [18]. In this regard, the OECD’s recommendations indicate that Chile should develop more systematic monitoring for cancer control: (1) extending the registry to more regions; (2) expanding the information collected from screening and diagnosis, similar to childhood and cervical cancer where data are linked to public and private sector providers; and (3) using PREMs and PROMs to improve the quality of cancer care and overall care [19].

PROM surveys are standardized and validated surveys that measure the results reported by patients during the perioperative period to know the perceptions of health status, level of perceived deterioration, extent of disability, and level of health-related quality of life, and can be classified as generic or specific to a disease [20]. For example, the QLQ-C30 questionnaire is generic for cancer, is available in more than 100 languages, including Spanish [21], and is the most widely used questionnaire [22]. On the other hand, the QLQ-PAN26 questionnaire [23] is specific for pancreatic cancer. These 2 questionnaires can be used together. The use of PROMs in clinical practice is associated with (1) reduction of emergency care; (2) improved doctor-patient communication, quality of care, quality of life, and experience with providers; and (3) better survival compared with usual care in patients with metastases, who are undergoing chemotherapy [24]. Other studies suggest that routine PROM collection may improve quality of life and outcomes for pelvic cancer patients [25], identify undetected symptoms [26], and aid in the clinical management and intervention of adverse effects [27].

However, the quality of life of people with cancer is influenced by not only the complications of the disease, but also the consequences of treatment involving a high cost [28]; therefore, it is important to include the measurement of financial toxicity in PROMs, an example being the COST-FACIT survey [29], which has 12 questions divided by themes (affect, coping, family, financial, and resources) [30].

In general, the use of standardized surveys allows comparative studies to be carried out; however, it is also possible to create surveys that adapt to the local reality. For this, it is recommended to follow the ISOQOL PROM measurement standard [31,32]: (1) Conceptual model: description and framework; (2) Confidence: degree to which the measurement of the patient-reported outcome (PRO) is error-free; (3) Validation: degree to which the instrument measures the PRO concept it intends to measure; (4) Interpretability: ease of understanding the meaning of the score of a PRO measure; (5) Minimum important difference: minimum score difference that patients or guardians perceive as important, beneficial, or harmful; and (6) Load: time, effort, and other demands on those who use the instrument or those who administer the instrument (investigator or administrative).

The collection of PROMs through digital means is known as ePROM. Authors have pointed out [33] that these have greater acceptance and preference by patients, lower costs, similar or faster completion times, and better data quality and response rates, and that patient management of symptoms is more appropriate. The disadvantages identified are related to privacy, large initial financial investment, and the digital divide in the case of some people.

With regard to PREM surveys, which are surveys of patients to collect information regarding lived experiences during care, the analysis of the impact of the care process [20], such as waiting times and doctor-patient communication, can be classified as (1) relational: regarding the relationship with those who provide care (an example of a questionnaire is CARE) [34] and (2) functional: with respect to practical situations such as availability of care [20].

The National Cancer Program of the United Kingdom’s National Health Service developed a PREM survey to monitor progress in cancer care, drive quality improvements, support cancer care commissioners and providers, and inform the work of the various charities and stakeholders supporting cancer patients [35]. In Chile, the PROM QLQ-ELD 14 (Spanish version), which measures the quality of life of older adults with cancer, has been validated, and it was concluded that the instrument in the applied population presented psychometric properties suitable for survivors of breast, colorectal, gastric, hematologic, lung, gynecological, head and neck, prostate, skin, and other cancers and found that gynecologic cancer survivors have the worst mobility [36]. Other investigators [37] used the QLQ-C30 and QLQ-STO22 questionnaires for stomach cancer and concluded that a significant proportion of patients showed an improvement in global health and perception of pain, despite the worsening of some symptoms that could be related to therapy, indicating that research is required on a large scale to confirm the observation.

The advantages of data collection using PROMs and PREMs justify the need for their continuous collection to increase the impact of an NCR. However, challenges need to be addressed regarding the need to collect indicators at a time when patients are comfortable (ideally at home [20]), and to design user-centered tools that enable user engagement to overcome digital divides [33].

### Technological Tools for the Collection of PROMs and PREMs

In the market, there are PROM and PREM collection tools that can be divided into generic and specific applications.

Form Builder applications are applications that allow the creation and distribution of generic surveys that, due to their functionality, can be used for PROMs and PREMs, namely Teamscope [38], Survey CTO [39], Beaver [40], KoBo Toolbox [41], REDCap [42], and ODK [43].

ePROM and ePREM applications are tools that have standardized surveys. Pro-CTCAE Patient Symptom Reporter has a web application for the registration of adverse events according to the CTCAE (Common Terminology Criteria for Adverse Events) [44]. Buddy Care Platform allows automatic sending of surveys and reminders, and incorporates instructions and educational material for patients [45]; however, it is only available for Germany, Finland, and the United States. Patient IQ [46] captures PROMs, identifies predictors of clinical outcomes, and improves the patient experience; however, it is available only in the United States. My Clinical Outcomes is a web application that allows regular collection of information on diagnosis, treatment, and any clinical condition [47]. Philips Quest Link integrates with external medical records and uses validated questionnaires to collect, process, and align PROMs and clinical information [48]. Heartbeat is a German web application that allows the collection and analysis of PROMs, and it can be integrated with external clinical records through the FHIR (Fast Healthcare Interoperability Resources) clinical message exchange standard [49]. Zedoc PROM, which is a platform for the integral management of PROMs, has integration with external systems through FHIR and has the support of Logical Observation Identifiers Names and Codes (LOINC) and Systematized Nomenclature of Medicine-Clinical Terms (SNOMED CT), and it is available for New Zealand, Singapore, and Australia [50]. Force Therapeutics has integration with the American Joint Replacement Registry and other records, reserving the right to use the information registered on the platform [51].

Form Builder applications are useful for the development of surveys; however, they are designed for projects in which it is not necessary to have an integration. On the other hand, specific applications provide more functionalities than necessary, have associated payment for their use, and are not available in Chile, and currently, some reserve the right to use the data collected.

It can be said that from the technical point of view, there are advances that allow the development of technological tools for the collection of PROMs and PREMs, but from a patient-centered approach, it is essential that this development contemplates the needs of primary users, that is, patients and cancer professionals.

### Research Goal

This work aims to present the results of a qualitative case study aimed at identifying the needs of cancer patients during the breast cancer treatment process in order to design a mobile app that allows the reporting of adverse symptoms and impacts, and that improves the quality of life. In particular, the research questions addressed in this study are as follows:

1. What information does a patient need during the breast cancer treatment process?
2. What information does a health care professional need from a patient during the breast cancer treatment process?
3. What are the user roles that must interact with the application, so information needs are met?

To address these questions, we involved patients and health care professionals in a co-design process.

## Methods

### Ethics Approval

The research protocol was reviewed and approved by the Scientific Ethics Committee CEC Med-UC (3/2019).

### Design and Sample

A qualitative case study was conducted to address the research questions. Participants were informed about the study and voluntarily signed the consent document. The study was carried out between June and July 2019. Data were collected though focus groups and a survey. The methodology of focus groups was used to survey the needs of 2 types of users: breast cancer patients and health professionals. We report the qualitative study according to the guidelines in [52].

The focus groups were conducted by the first author (CT), who was the principal investigator of the research project and has more than 15 years of experience in eHealth research. There was no relationship with the participants prior to the study. Participants had knowledge about the goals of the research and acknowledged the researcher and the research team as participants of the NCR project. The methodological orientation of the research was content analysis, as we aimed to identify common information need themes across the participants.

The participants of the focus groups (both with patients and health care professionals) were selected by convenience and were the patients or professionals of the Chilean Health Institution. In all focus groups, the participants were approached face-to-face. Two focus groups were conducted with cancer patients (n=4). One participant dropped out without giving further reasons. All participants were women aged 34 to 53 years. The group of health professionals included those who participated in the care of patients and belonged to the cancer committee of the cancer center where the study was conducted (n=9). No health care professionals dropped out from the focus groups, and only 4 surveys were collected after the activity.

Data were collected in the clinic, and only the participants and researchers were present during the activity. The cancer patients were divided into the following 2 groups: stage I, II, or III patients who were undergoing chemotherapy (n=3) and stage I, II, or III patients who had finished chemotherapy between 3 months and 1 year (n=1).

For the patient focus groups, the following 4 guiding questions were used: (1) What information about your disease would you like to have? (2) What information regarding your treatment would you like to have? (3) In what instances would you like to have feedback from a health professional during your treatment? (4) What symptoms have you reported to your doctor that have been caused by an adverse effect? Field notes were taken by the third author (RN). The first focus group with patients lasted 55 minutes, and the second one lasted 33 minutes. In the 2 cases, data saturation marked the end of the interviews.

For the health care professional focus group, the guiding question was as follows: What are the information needs of cancer patients during treatment? At the end of the group session, a written survey form was delivered, which was to be sent later to the research team and which inquired about the information that professionals required from patients and how important and feasible it was to provide the information requested by patients through a digital tool.

**Figure 1:**
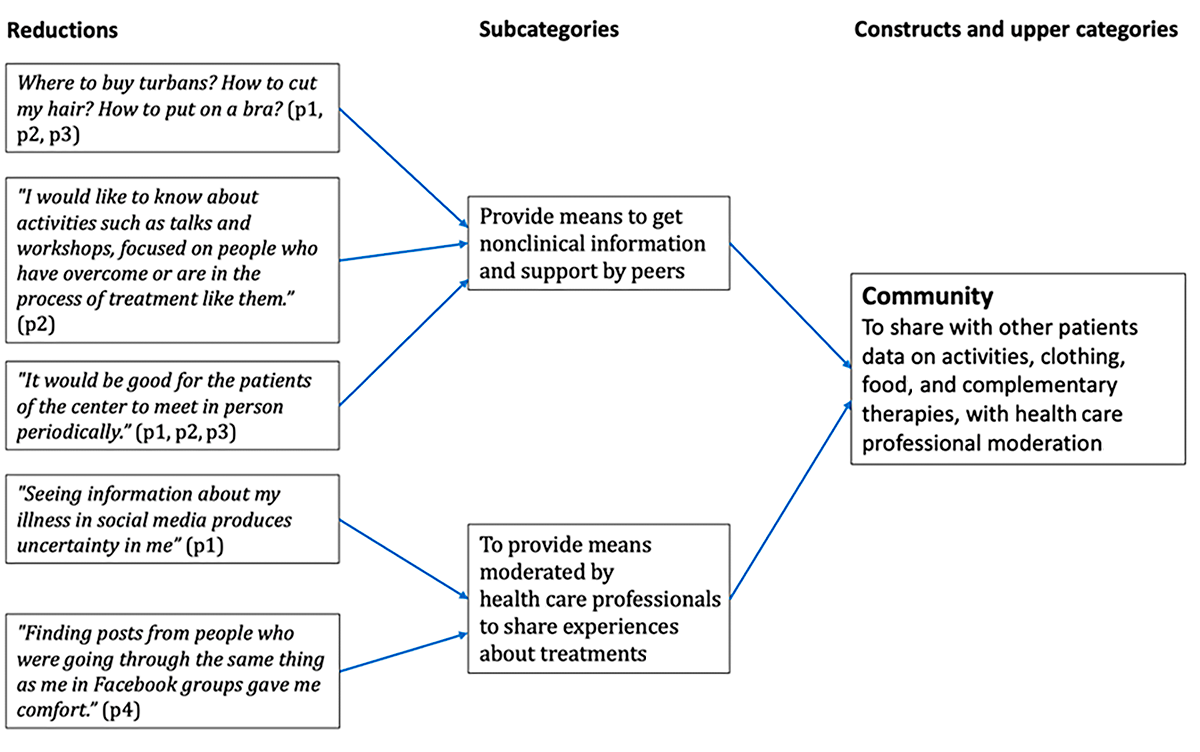
Example of the analysis process.

Two researchers analyzed the collected data. The data were analyzed following the content analysis method with an inductive approach [53]. One of the researchers coded the transcripts of the focus groups to obtain the subcategories. The abstraction process, that is, the grouping of the categories around less numerous and higher-level categories, was carried out jointly by the 2 researchers. The main categories emerged from grouping the generic categories into functional groups, which were then considered in the design process as modules of the system. In Figure 1, we provide an example of the content analysis process.

The results are presented as answers for each of the research questions.

## Results

### Cancer Patients

This group corresponds to patients with stage I, II, or III breast cancer who are being treated with chemotherapy or who have completed it. The information obtained from the guiding questions is summarized in Table 1.

**Table 1:** Summary of the information required by cancer patients.

Question	Scope of information required	Aspects pointed out by patients
What information about your illness would you like to obtain?	Disease overview	What is disease? Symptoms Tests Treatments Procedures Aftermath
What information regarding your processing would you like to obtain?	Treatment information	What is going to happen next? What is marking the tumor? How is the effect of the treatment determined? What can be done and what cannot? How much exercise can be done? Can other diseases affect cancer? How do I know if what I prepare to eat corresponds to a light regime? Are the sensations and consequences I have normal? Positive and negative effects of complementary therapies Activities focused on people who have overcome the disease or are in the same process Other domestic aspects that impact quality of life (Where to buy turbans? How to cut your hair? How to put on a bra?)
In which instances would you like to have feedback from a health professional during treatment?	Parameters of normality and abnormality in response to treatment	Help to identify what is expected and unexpected in the face of chemotherapy What are the symptoms for which the professional should be notified? What medicines to use in case of dizziness or discomfort?
What symptoms have you reported to your doctor that have been caused by an adverse effect?	Symptoms to report	What is the difference between adverse effects and those of the treatment? Symptoms that affect quality of life Vomiting Allergies

### Health Professionals

The group corresponds to health professionals who care for cancer patients participating in the study. One aspect that stood out in the focus groups with professionals was that they considered it necessary to provide patients with guidance on the administrative and financial process in which they are immersed. They also mentioned the need to have the clinical information of other professionals that patients consulted in the process of research, diagnosis, and treatment of the disease. In addition, all professionals who responded to the survey pointed out that it was important to provide patients with generic information on symptoms and intensity that should be reported by the patients with different levels of urgency and provide information on daily activities for well-being (diet and physical exercise). Most professionals pointed out that it was important to respond to private clinical questions (chat or private message) and public questions (forum or social network) of patients through a digital means of communication and deliver nonclinical data for the well-being of cancer patients (activities, information on turbans and bras, etc). On a scale of 1 to 5 (where 1 indicates little disagreement and 5 indicates strong agreement), the importance of providing information on complementary therapies to clinical treatments was scored 5 by a professional, and the other 3 professionals scored it 3. In the responses, the majority of respondents indicated (scores 4 and 5) that actions qualified as important were feasible, except for the item of answering private clinical questions, where all professionals scored it 2 or 3. On the other hand, among 12 symptoms presented to determine the adverse effect of a treatment, all professionals agreed on one, pain, as an important symptom to report. Likewise, all professionals who responded to the survey agreed that for adverse effects, it is necessary to know the temporality, severity, and intensity.

### Proposal for the Design of the App

From the collection of information with patients and cancer professionals, 4 areas of information needs were detected: (1) knowledge regarding the disease in general, (2) feedback for the reporting of symptoms, (3) support in administrative processes and (4) complementary information. The solution proposal consists of a mobile app called +*Contigo* (Spanish word meaning +With you), whose functionality will be described considering the 4 modules that compose it, according to the actors involved. Based on this, the prototype design for the main user interfaces and the design of the system architecture, including its components and deployment, will be shown.

#### Modules

**Figure 2:**
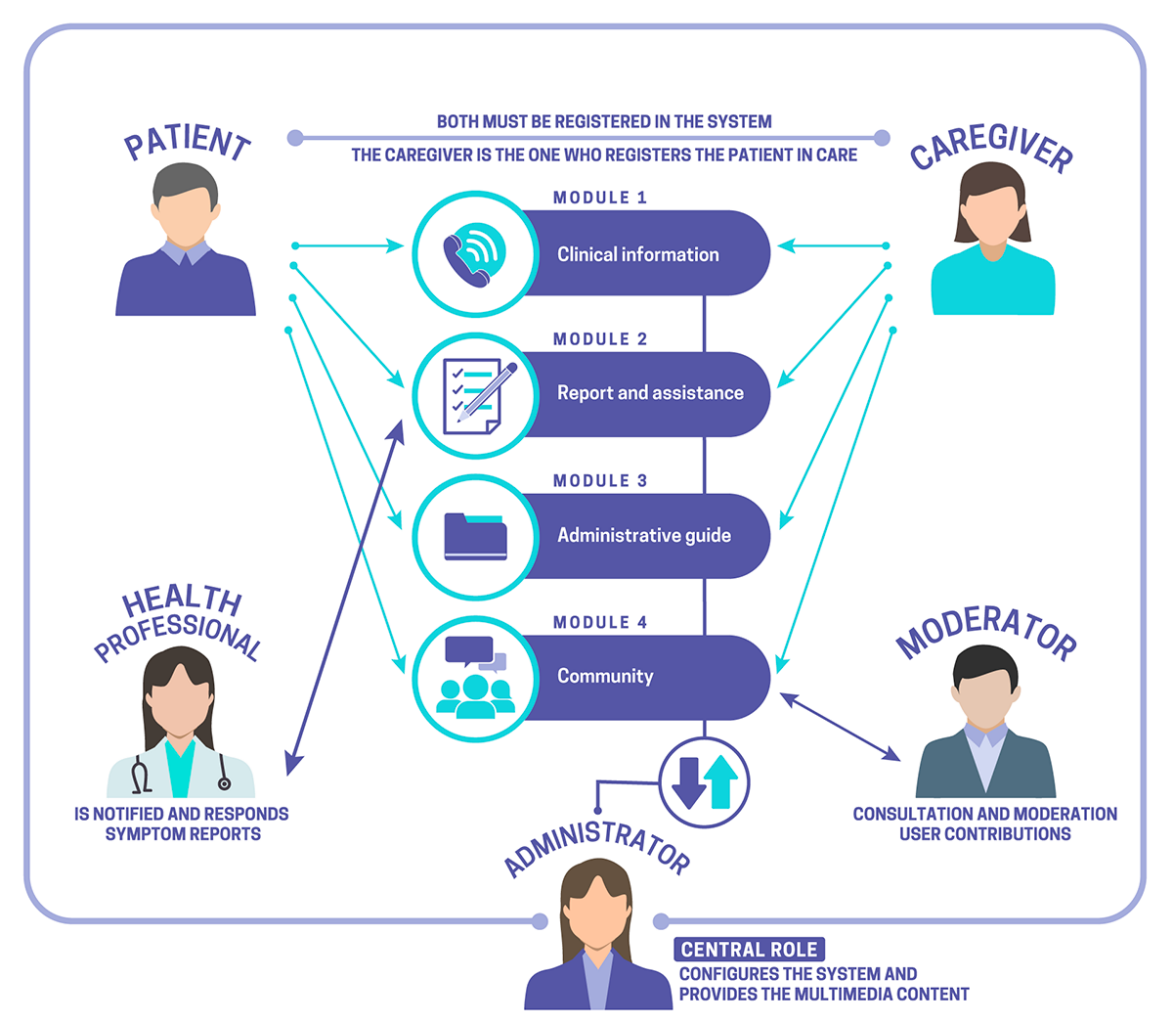
+Contigo app diagram.

The solution in *+Contigo* proposes 4 modules with different modalities of use: (1) Clinical information, (2) Report and assistance, (3) Administrative guide, and (4) Community, which are oriented to different actors or types of users. It is considered that a large part of the actions will be carried out during the process of implementation of the solution (pilot period and white gear). Figure 2 shows the diagram of the +*Contigo* app.

Actors can access the 4 functional modules of *+Contigo*. Both users are required to register in the system, and caregivers are required to register the patients under their care. Although the system will know the user’s identity, it will be protected for any interaction with other actors in the registry, unless the user explicitly authorizes its use.

The mobile actor will be involved only with the module and complementary information unit and will be able to contribute information to the discussions of the patients’ and caregivers’ community, as well as edit or delete published information that may be considered harmful to users from a clinical or quality of life point of view.

The professional actor of the avalanche will be directly related to the reporting and assistance module and will receive notifications from patients, which will be classified by priority and severity, being able to deliver online responses (audios, texts, images, videos, or reference links) or other types of actions outside the system (telephone contact, schedule regular control, or schedule urgent control attention).

The administrator has a central role in the +*Contigo* management process consisting of configuring the system and providing multimedia content, with the ability to define the different levels in the clinical information module; configuring the different types of symptoms, the degrees of severity with their description, and the alert priority in the module of support and the system; and uploading multimedia content, adding questions, providing answers, adding checklist items in the module of administrative support, and generating discussion topics in the module of the community.

**Figure 3:**
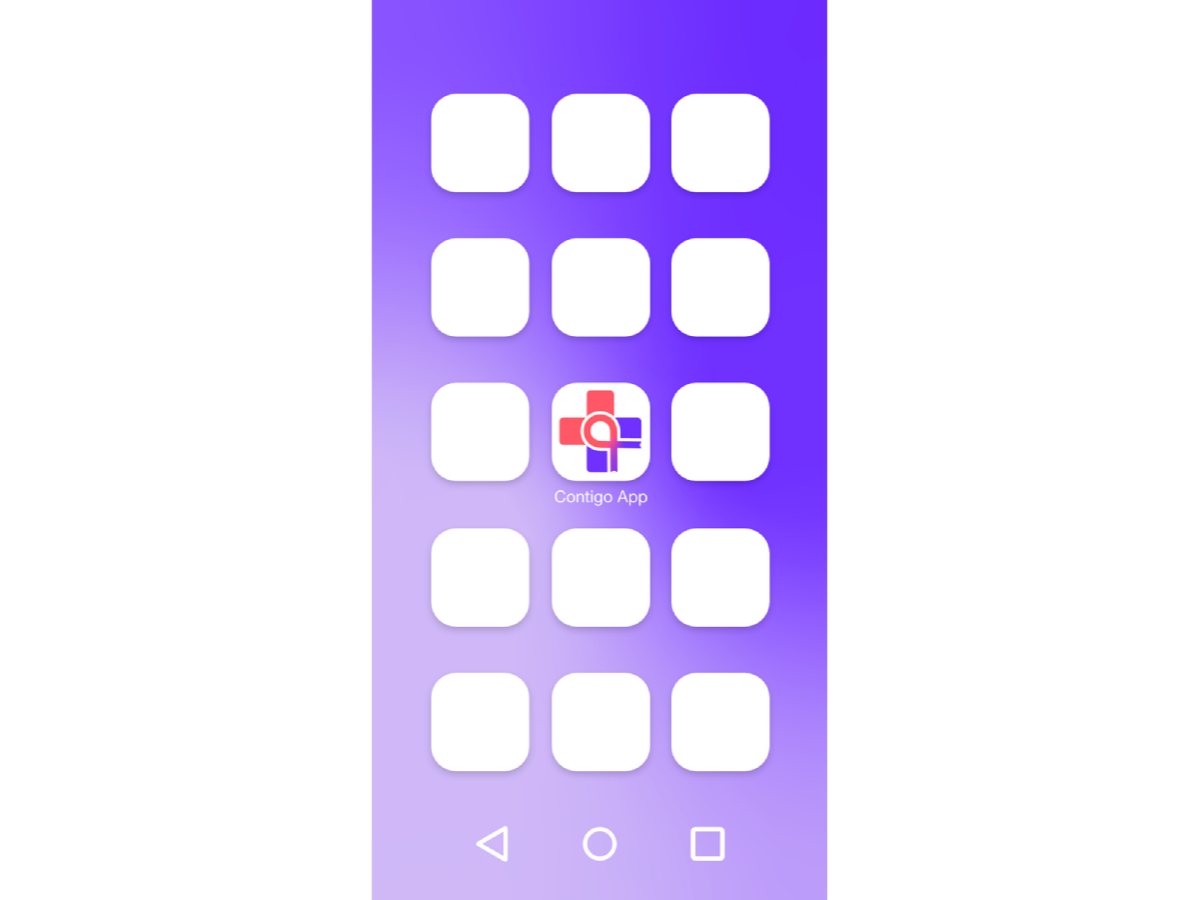
Main page of +Contigo.

On downloading the app on a mobile device, users will be able to recognize *+Contigo* (Figure 3), with the logo on the screen of the mobile device.

**Figure 4:**
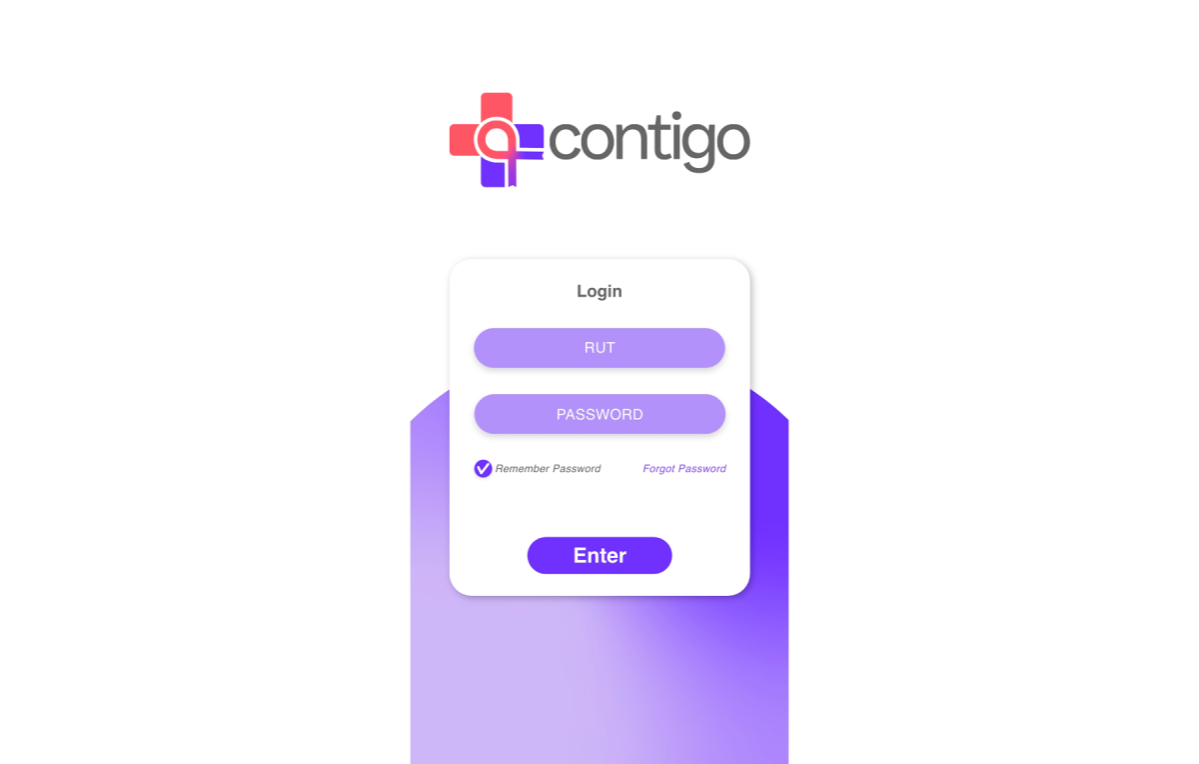
+Contigo login screen.

When entering the app, the user will see the login screen (Figure 4) to start a secure session, which is protected by a password and a chosen username.

**Figure 5:**
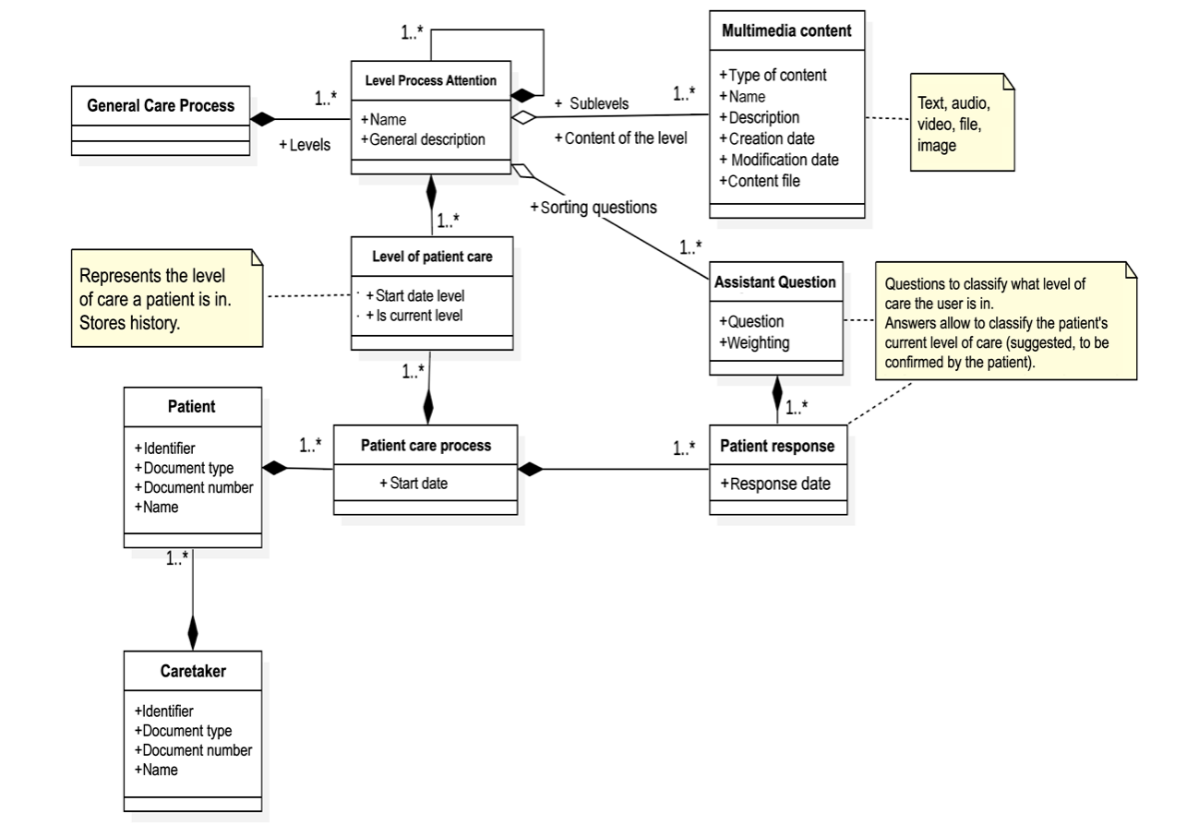
Clinical information module. The image shows with dependence (black rhombus) or without dependence (white rhombus) and with many relationships (1.*) or with possible relationships (0.*).

**Figure 6:**
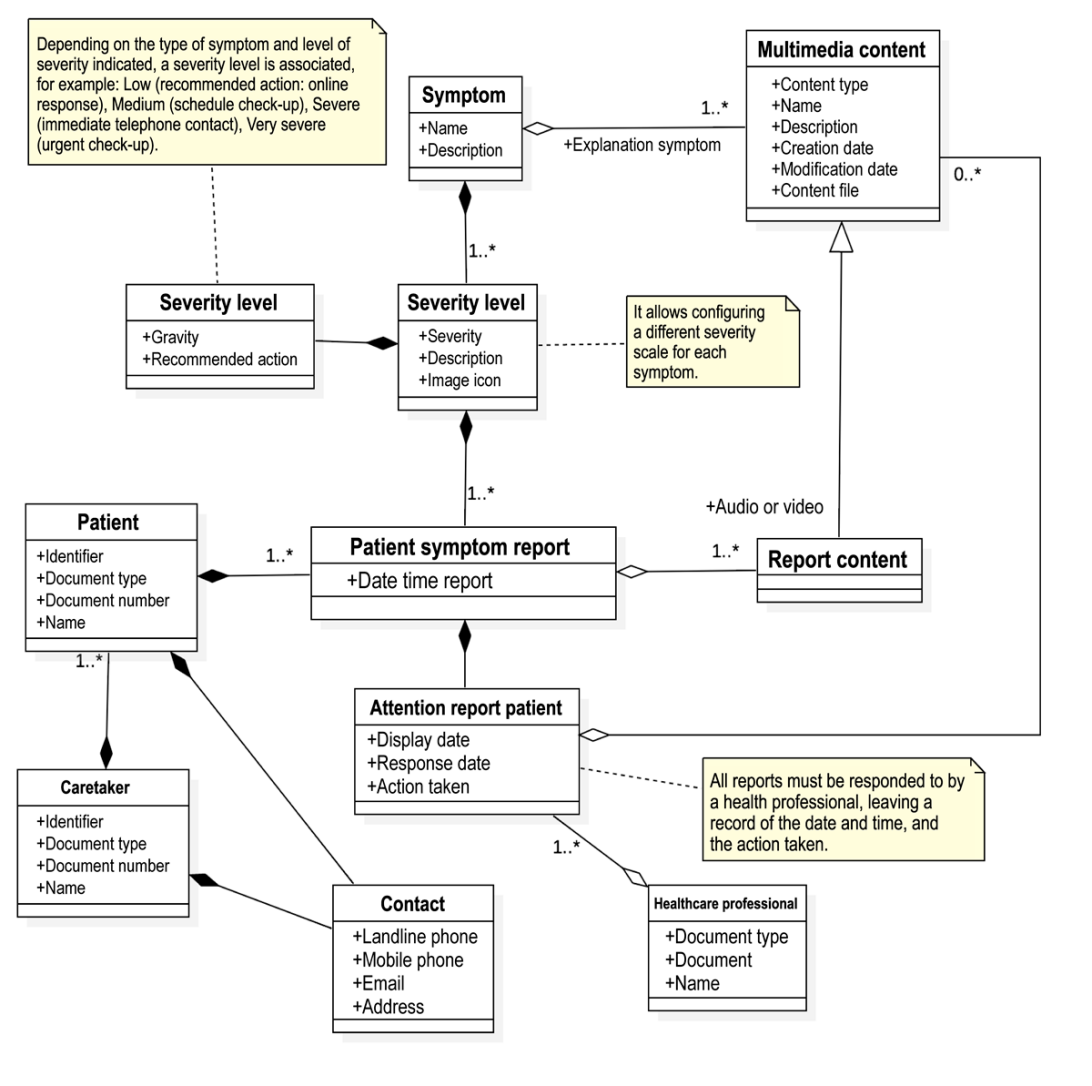
Report and assistance module. The image shows with dependence (black rhombus) or without dependence (white rhombus) and with many relationships (1.*) or with possible relationships (0.*).

**Figure 7:**
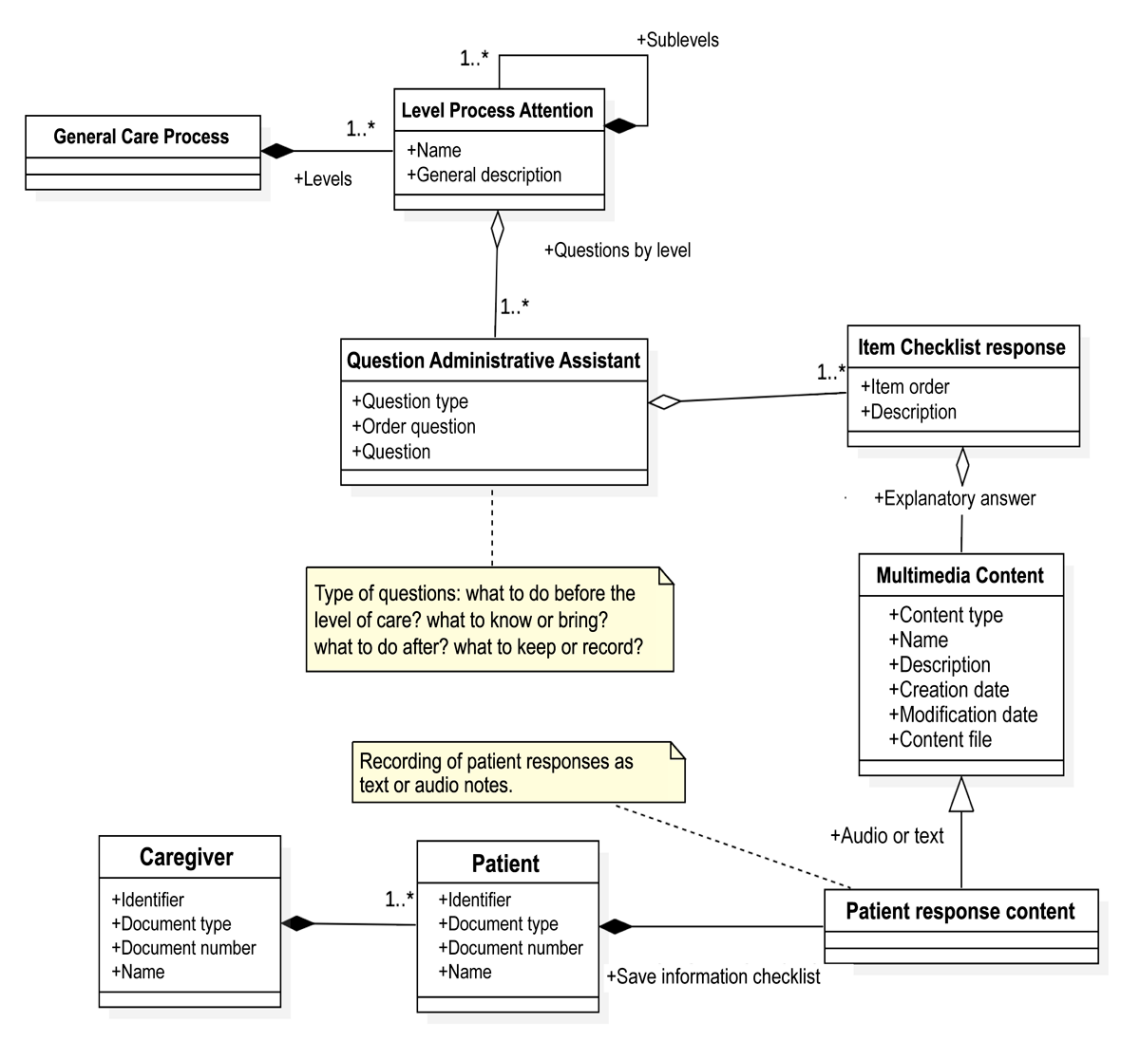
Administrative guide module. The image shows with dependence (black rhombus) or without dependence (white rhombus) and with many relationships (1.*) or with possible relationships (0.*).

**Figure 8:**
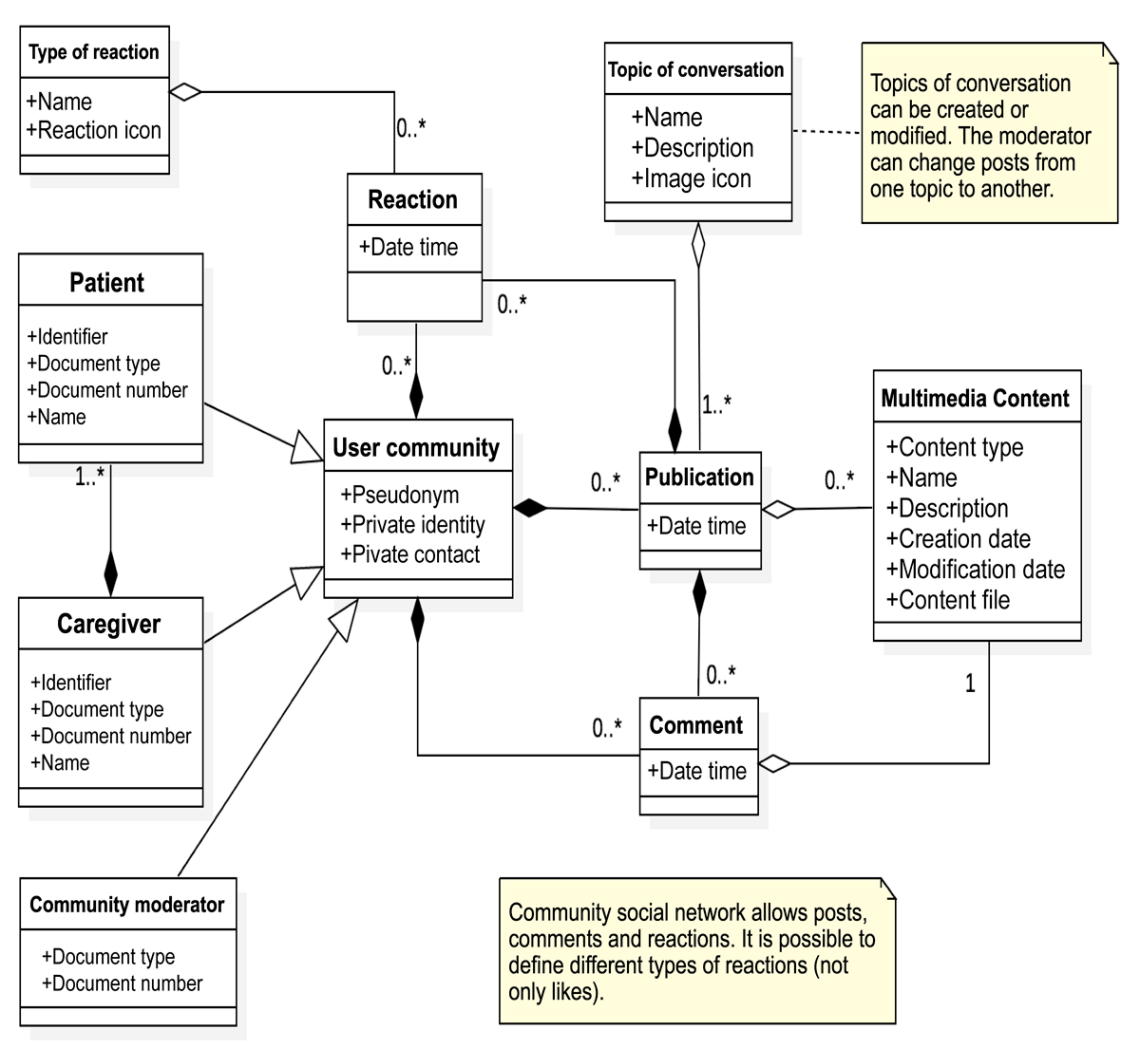
Community module. The image shows with dependence (black rhombus) or without dependence (white rhombus) and with many relationships (1.*) or with possible relationships (0.*).

Figures 5-8 present the domain model of each module. This conceptual modeling represents the vocabulary of the system, as well as the relationships allowed between these concepts, with dependence (black rhombus) or without dependence (white rhombus) and with many relationships (1.*) or with possible relationships (0.*). Added text boxes with appended notes (dotted lines) that complement the information are delivered to clarify how the model will respond to certain functional requirements.

The clinical care process assistant module (Figure 5) provides information to the patient regarding the level of care in which the patient is, using questions and answers that allow the patient to internalize the disease, the phases of the process, the treatments, and, in particular, chemotherapy.

The report and assistance module (Figure 6) allows the user to consult detailed information on chemotherapy symptoms, indicating levels within expected ranges and those that are out of normality, and allowing symptoms to be reported based on a scale of type and severity. This module enables health care professionals to interact with patients via text, audio, or video messages, or initiate a phone call to the patient’s mobile.

The administrative guide module (Figure 7) is an informative module with a focus of questions and answers, together with a checklist to guide the patient regarding the eventual and possible main procedures to be carried out in the process of the disease, which is associated with the different stages and substages of the care process.

The community module (Figure 8) provides practical and everyday information for nonclinical aspects. This module is aimed at supporting the quality of life (eg, where to find clothing, support groups, and information and dissemination activities); posing as a social network, where users can use a fictitious or real name, with the objective that the community shares the information it considers necessary and relevant; and providing links to external resources, comments, photographs, and audio or video messages.

#### Interface

A prototype design for the main user interface and the design of the +*Contigo* architecture are presented, including its components and deployment to finally propose the data model.

**Figure 9:**
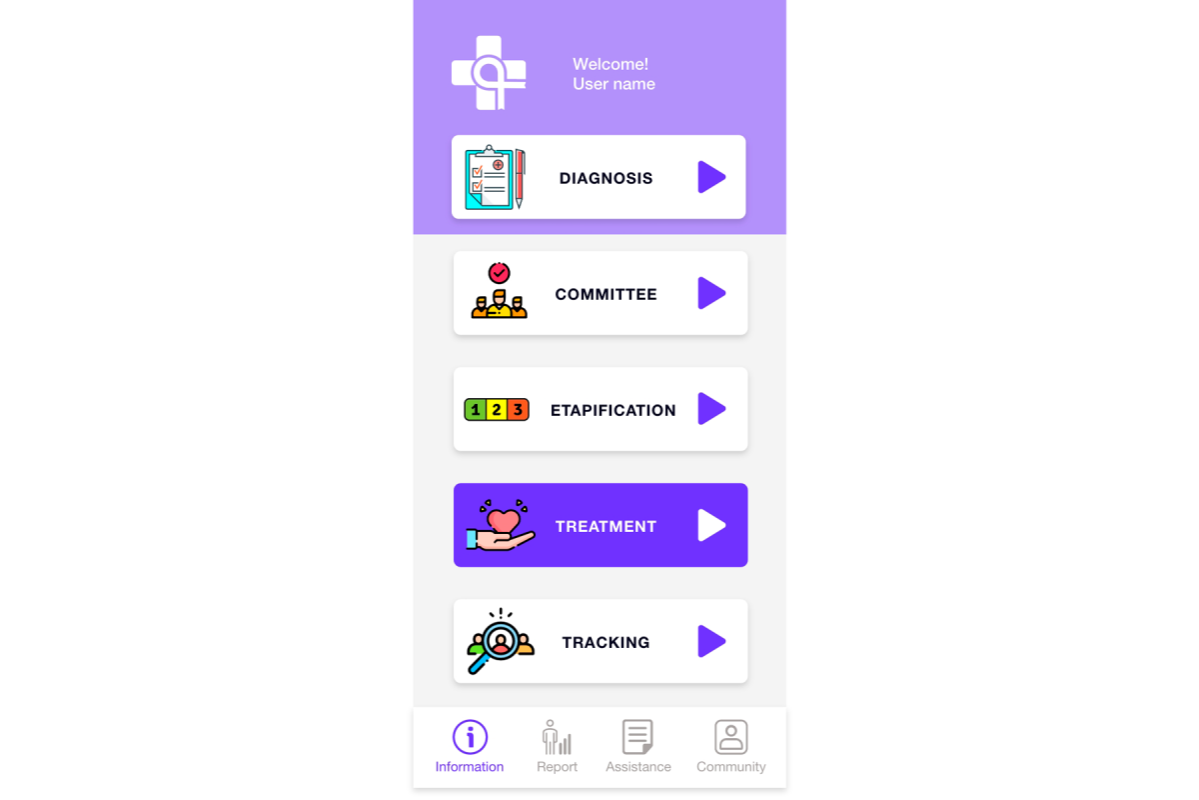
Main view of the clinical care process (first level). The selected section is identified by a color change.

With the main version of the first level of the clinical information module (Figure 9), the user can access information on the whole process, patient diagnosis, multidisciplinary case analysis, cancer staging, treatment, and case tracking. From the bottom menu, the user can quickly access information and other modules such as port, administrative guide, and community.

**Figure 10:**
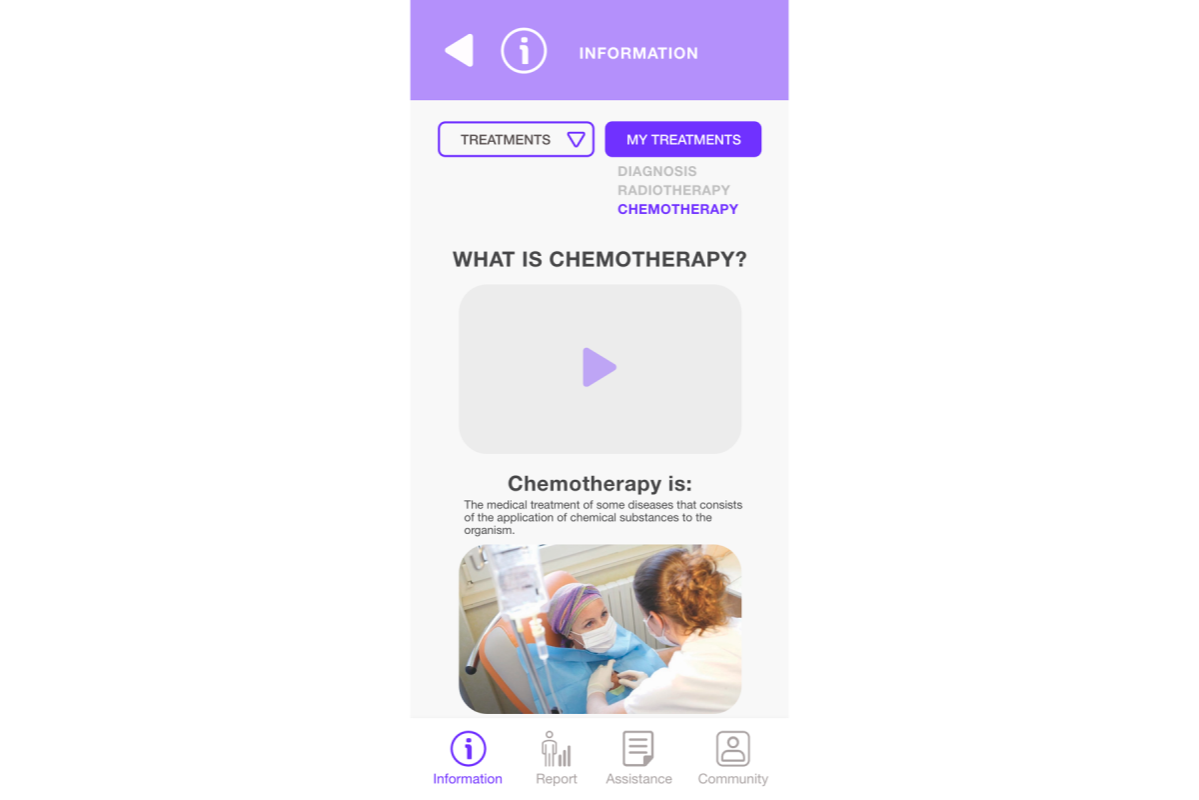
Clinical care process assistant module (sublevel).

The sublevel of the clinical information module (Figure 10) displays multimedia information (text, audio, and video) of the stage of the clinical care process selected in the main view (in this case, Treatment), where the active section of the interface in which the user is located is identified through a color change.

**Figure 11:**
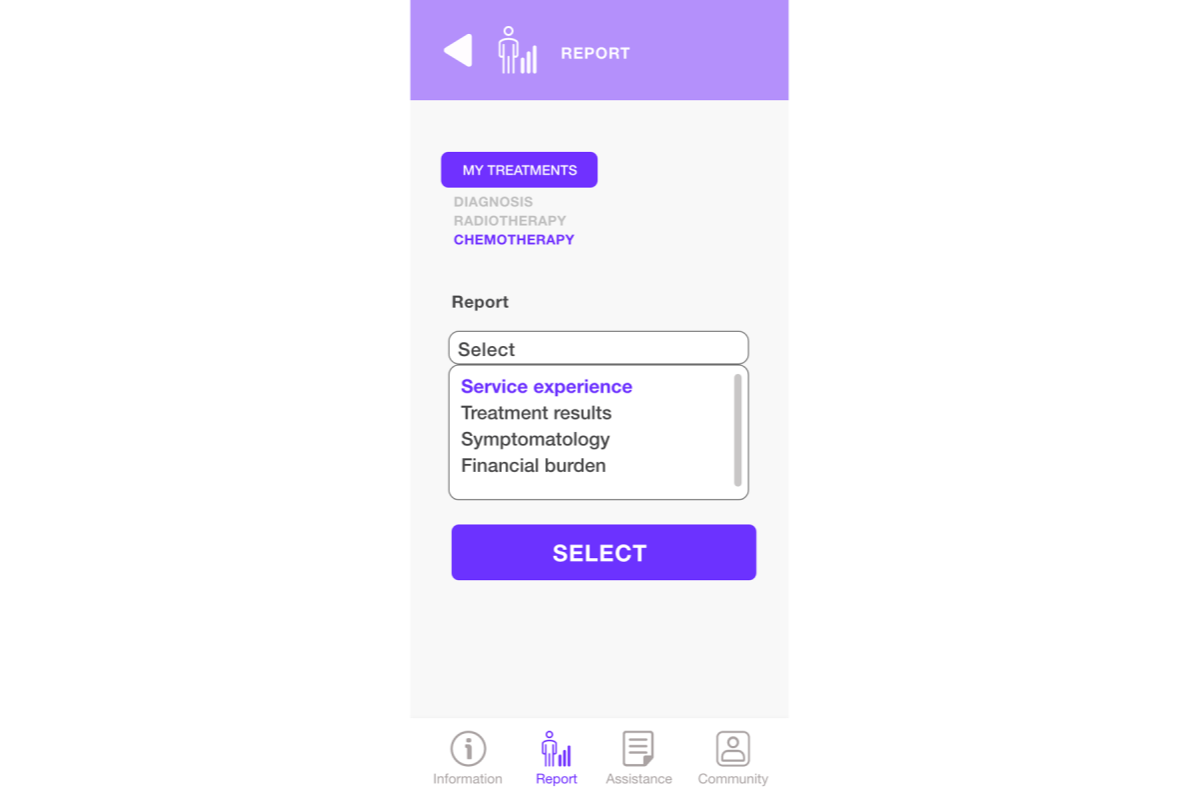
Report and support module parameters.

The report and assistance module (Figure 11) is accessible directly from “Report” in the bottom menu. Here, the patient can choose what aspects they want to report (health outcomes, care experience, symptoms, or financial burden).

**Figure 12:**
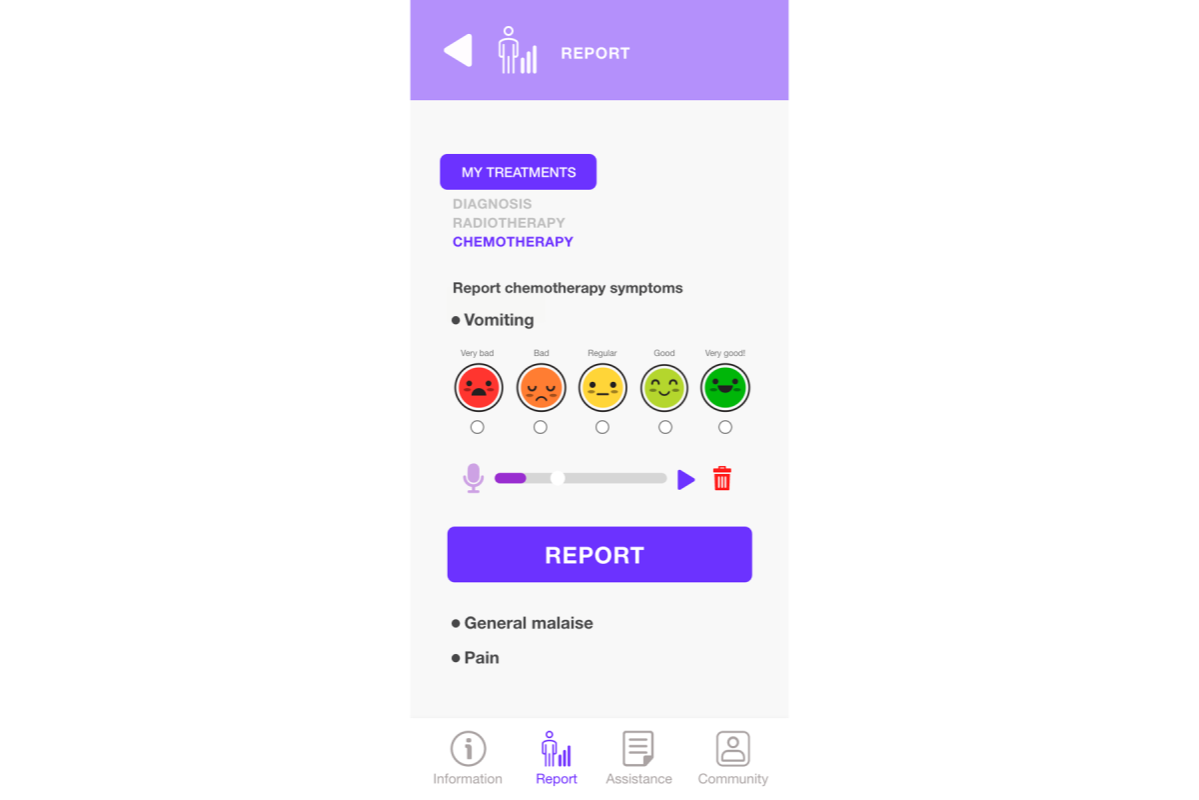
Symptom report and assistance module.

The report of symptoms related to the disease or treatment (Figure 12) is made by indicating severity through a scale represented in a set of 5 selectable faces (radio button) that can be accompanied by complementary information through a voice message by pressing the microphone icon, which activates this function on the phone.

**Figure 13:**
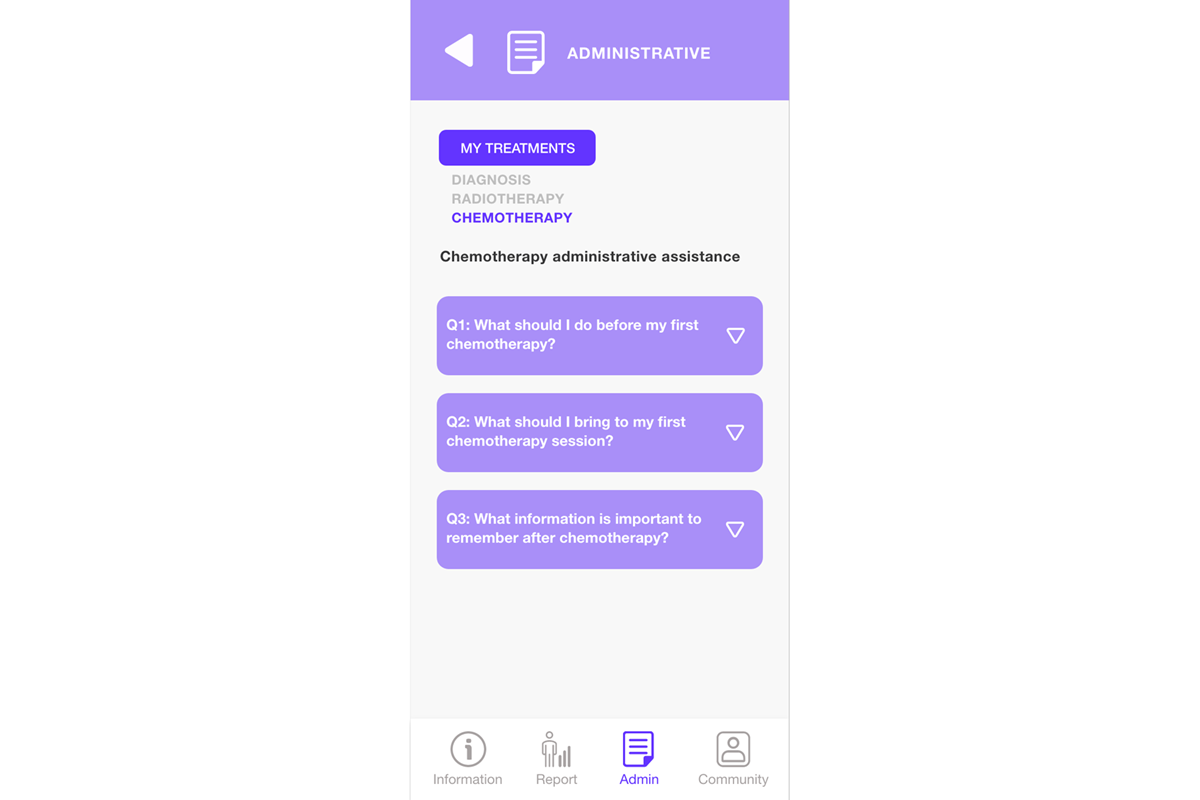
Administrative guide module (assistant questions).

Answers to frequently asked questions in the administrative guide module (Figure 13) are available directly from “Assistant” in the menu below. Here, administrative information is provided using the approach questions, response, and checklist to guide the patient regarding the main procedures to be carried out. When a question is selected from the list, the answer is displayed, and when it is selected again, it collapses, hiding the answer.

**Figure 14:**
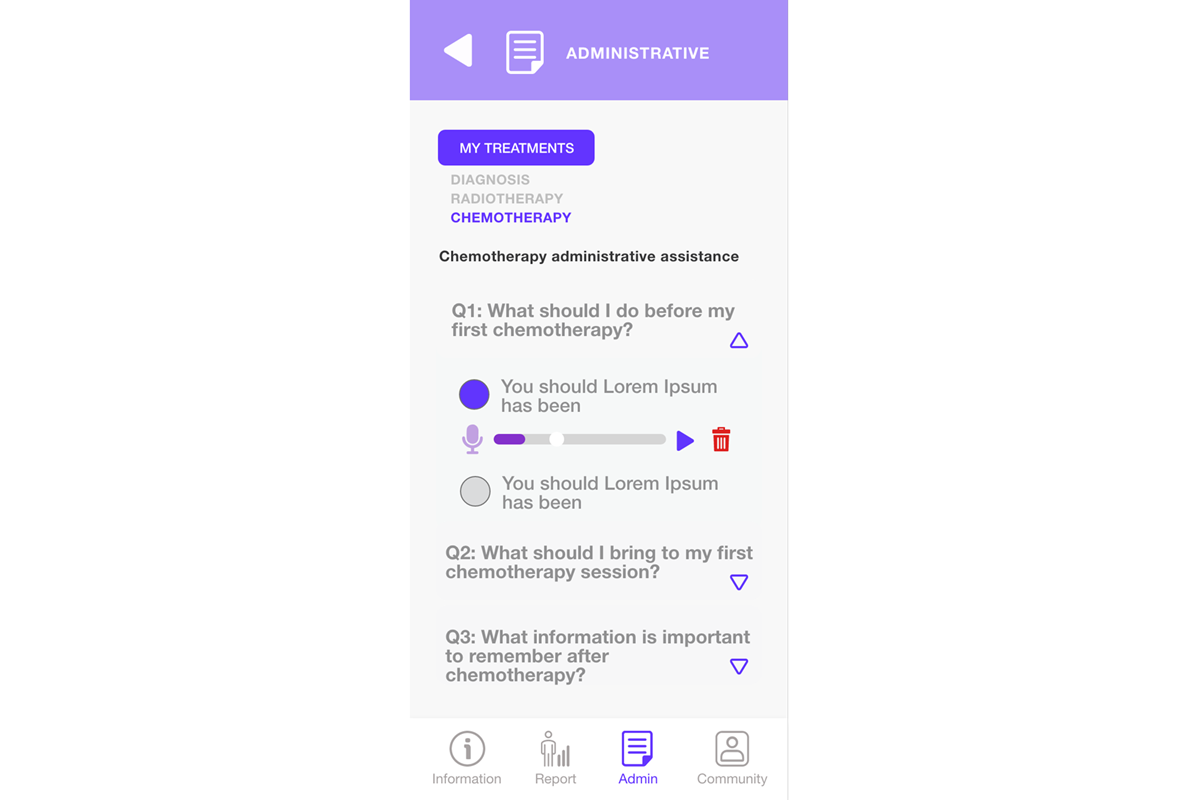
Administrative guide module (record of patient voice notes).

The recording of voice notes of the patient in the administrative guide module (Figure 14) is accessible directly from “Assistant” in the lower menu, showing the answer to a selected question in this case, which corresponds to a list of steps, among which the patient can identify those that were fulfilled or not. In addition, an audio can be incorporated into each response by pressing the microphone icon.

**Figure 15:**
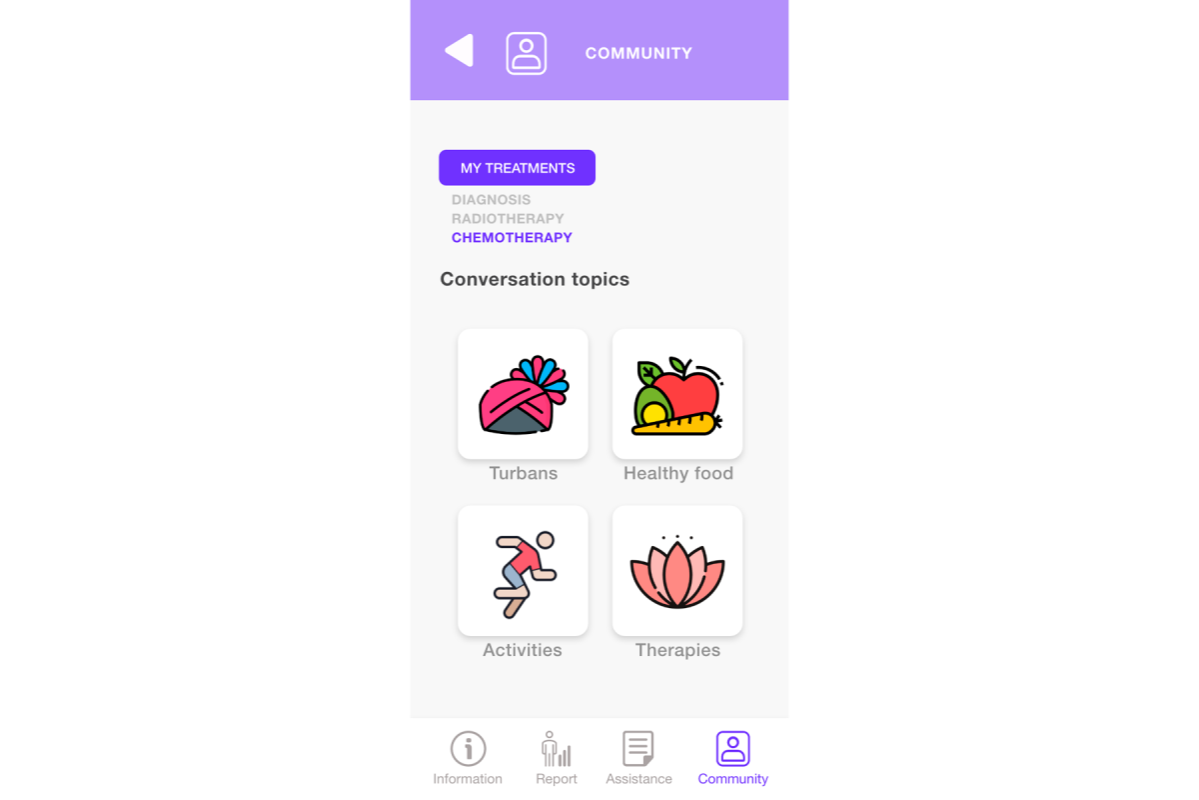
Possible topics of conversation in the community module.

Figure 15 exemplifies some possible topics of conversation in the community module, which is accessible directly from the bottom menu. The interface groups several categories, and each has different conversation topics that can vary, renew, and expire, depending on the interests of the participants. The functionality of this section is like a conversation chat, but a moderator (health professional) has been put in charge of reviewing the material before its publication to ensure that the suggestions and recommendations to be discussed in the community do not affect the quality of life of the participating patients.

#### Architecture

A 3-layer architecture is proposed for the development of the app, considering microservices, that is, monolithic apps (with their self-contained server), and with the scope limited to a subset of the data model (consistent with the concepts of the domain model). To ensure portability, the development of a progressive web application compatible with different mobile operating systems (Android and iOS) is proposed, with development in Angular 8. It is proposed to use the same server and database management system that the organization already has to implement the data tier. The main architectural definitions are specified in Table 2.

Table 3 shows the quality attributes that the app must have.

**Table 2:** Definitions of high-level architecture.

Concern	Solution
Type of application	Stand-alone web application
Relevant technologies	Ionic 4, Angular 8, Postresql (or available), Java 7
Deployment strategy	Containerization
Cross-cutting concerns (optional)	N/A^a^
Styles and patterns of architecture	Microservices, Model-View-Controller design pattern
Reuse, purchase, or construction	Construction

^a^N/A: not applicable.

**Table 3:** Quality attributes of the app.

Quality attribute and decision		Element	Justification
**Compatibility**			
	Monolithic Java app	Spring boot	Development of self-contained monolithic apps on the Java framework (spring boot) for server independence.
	REST API^a^	REST API consumption of patient identification and contact information	Interoperability standards are not implemented due to not sharing clinical information.
**Reliability**			
	Circuit Breaker design pattern	Using Circuit Breaker design pattern	The Circuit Breaker pattern is used to detect faults and encapsulates the logic of preventing a failure from constantly repeating during maintenance, including a temporary system failure or unexpected system difficulties.
**Safety**			
	Spring boot	Spring boot security	The security component of the development framework will be used to provide security to the back end.
	Angular 8	Angular security	The security component of the development framework will be used to provide security to the front end.
**Maintainability**			
	Microservices	Microservices architecture	Minimum data models will be defined for each module, with separate back end apps for each.
**Portability**			
	Ionic 4 - Angular 8	Angular utilization 8	The use of Ionic 4 allows the user to port the app to different mobile operating systems. Angular 8 supports multiple browsers (Chrome, Firefox, Edge, and Opera).
**Performance efficiency**			
	Query tuning	Make query adjustments and use of indices	Make adjustments to the database and queries, and apply the necessary indexes.
**Usability**			
	Angular 8 and Angular Material	Use of Angular 8 and Angular Material	The use of these technologies allows users to achieve their goals effectively, efficiently, and with satisfaction in a specific context of use.

^a^API: application programming interface.

## Discussion

### Requirements for Reporting Health Experiences

While various applications for reporting health experiences and outcomes are available, some are general [38-43], which can be adapted for PREM and PROM purposes, but do not allow integration, and others are specific [44-51], but provide more functionality than necessary, have associated payment, and are not available in many countries, with some reserving the right to use the collected data. In this sense, our study managed to identify 4 categories of requirements, generating a mobile app design based on the responses of primary users (patients and cancer professionals). Although the sample corresponded to patients with breast cancer, it is estimated that *+Contigo* would be useful for patients with other types of cancers.

It is currently recognized that cancer is not only a public health problem, but also a sociohealth, social, and economic problem, which affects the patient, family, and community [4]. Therefore, it is necessary to know the demands and needs of patients and carry out all actions that tend to reduce waiting and ignorance of this disease, given that many patients have a delayed diagnosis (23.57%), being largely the cause of health system inefficiencies (79.03%) [54]. New technologies can be a means to bridge inequity gaps [55] and a means to access the information that patients and clinicians need, and safeguard the methods, standards, processes, and tools that have been reported in the literature to assess the quality of health information systems [56,57].

### Threats to Validity

According to Wohlin et al [58], we analyzed the threats to the study’s internal, construct, and external validity. Internal validity refers to the existence of other elements affecting the observed results. As in any case study, the specific context (in this case, the cancer center) might affect the results. Nevertheless, our approach is not explanatory but exploratory, so the results are meant to be interpreted in the context of the case study provided in the Introduction. Construct validity deals with the degree to which the measurements reflect what the researchers have in mind and help answer the research questions. We addressed this threat by designing the questions for patients and health professionals beforehand and explaining them to the subjects during the focus groups. The survey questions were designed to answer research questions 1 and 2, while the third research question came up from the requirements analysis. External validity addresses to what extent it is possible to generalize the findings. The sample size and the scope of the study certainly limit the generalizability of the results. However, as in other case studies, we intend to provide enough detail for the audience to extend the results to cases with common characteristics and for which the findings are relevant [59]. Finally, the conclusion validity in case studies mainly deals with the reliability of the measures. We addressed this threat by providing details of the content analysis and the categorization of the findings; however, different researchers might come up with different categories by following the same analysis procedure.

### Usability Evaluation

We evaluated the app’s design through a heuristic evaluation [60], where trained evaluators identified potential usability problems by reviewing the app or prototypes through the application of a set of design best practices called heuristics. Three information system and user interaction design experts from Chile and Spain performed the assessment. The evaluators reviewed the front end of the patient and caregiver mobile app and mockups of the apps for the health professional and social network moderator. The evaluators received a high-level description of the app features (described in the Modules section of this paper) and the app prototypes. The evaluators were asked to base their review on the heuristics for mobile apps by Bertini et al [61]. The reported problems indicated the function and role of the user, the heuristics involved, and their severity, which was classified as low, medium, and high. Evaluators were asked to provide recommendations for the improvement of each. 

A total of 62 usability problems were identified by the experts, and 18 of them had high severity. Among the most severe issues, the more compromised heuristics were “Ease of input, screen readability, and glanceability” (7 issues), “Esthetic, privacy, and social conventions” (6 issues), and “Consistency and mapping” (5 issues). The features with more severe issues were patients’ symptoms and experience with reporting. Table 4 presents the features with high severity problems.

**Table 4:** Features with high severity issues.

User	Feature	Issues (high severity)
Patient	Home	2
Patient	Disease information	2
Patient	Report symptoms	4
Patient	Report mental health	2
Patient	Report quality of life	1
Patient	Report experience	3
Health professional	Home	1
Health professional	Reports	1
Health professional	Alert	1

Some of the most severe issues were related to the patient home screen and the disease information features, which was named “My Journey” in the app. The “journey” feature name and metaphor were directly elicited from the focus groups since patients wanted to avoid seeing cancer or disease in a daily use app. Reviewer 1 from Spain mentioned that the “My Journey” feature was not intuitive for finding information about the disease and treatment. We think this is due to idiosyncratic differences between Chile and Spain, and is consistent with the heuristic “Esthetic, privacy, and social conventions” in [61], which enforces taking into account the social and emotional aspects of the system. Regarding the results and experience reporting features, both Reviewer 2 and Reviewer 3 reported issues related to the heuristic “Ease of input, screen readability, and glanceability.” Given* *the length of the questionnaires, the reviewers recommended separating them into subsections, which would provide visibility on the questionnaires’ completion status, and autosaving the responses to avoid information loss. Regarding providing assistance for patient queries on the normality of their symptoms, Reviewer 1 and Reviewer 3 reported that the system should clearly separate the interaction with an automatic assistant or a health professional.

After the evaluation process, the researchers analyzed the problems graded with high severity. The evaluators’ recommendations for these problems were studied and grouped by the compromised heuristics and the associated features. From the information requirements identified in the case study and the heuristic evaluation, we present a set of guidelines for designing a mobile app for self-reporting results and experiences among breast cancer patients (Table 5). The guidelines consider the information requirements that must address the app and the usability considerations.

**Table 5:** Proposed guidelines for designing a mobile app for self-reporting results and experiences among breast cancer patients.

Information requirement guideline	Usability guideline
Disease overview information	1. Name the features that provide information about the disease matching patients’ social conventions.
Treatment information	2. Consider the actual treatment and status of the patient to avoid cognitively and emotionally overwhelming the patient. 3. Organize the information matching the timeline and flow of actions for the patient.
Treatment information (day-to-day affairs – community)	4. Provide interaction mechanisms for patients and community moderators to avoid disinformation and inappropriate social behavior.
Parameters of normality and abnormality in response to treatment	5. Patients should know whether their queries will be addressed by an automatic assistant (chatbot or other) or a health professional.
Symptom report	6. Apply the best usability practices for questionnaires: Break up questionnaires into multiple steps, provide completion status feedback, and autosave. 7. Provide prioritized notifications with contact information for health professionals to reach patients reporting abnormal symptoms.

### Conclusions

This study has allowed the characterization of the needs of the primary actors involved (patients and cancer health care professionals). The findings show that the main information needs are related to generic information on symptoms and intensity, which should be reported according to different levels of urgency; guidance on the administrative process; nonclinical data that contribute to the well-being and daily comfort of patients (activities, data on turbans and bras, etc); and daily activities for well-being (diet and physical exercise). The satisfaction of the needs must be supported by different mobile app features, including multimedia information on the disease and treatments, interactive forms and query mechanisms to report symptoms, experiences and requirements of assistance, and a social network for enabling community support for day-to-day affairs. The information needs require the collaboration of the community of patients, caregivers, health care professionals, and social network moderators. The heuristic evaluation of the user interface reveals that the app must consider delivering disease and treatment information taking into account the emotional effect on patients as well as their social conventions, and address the length of some questionnaires (particularly symptoms and experiences) with usability best practices.

Future research will focus on empirically assessing the effects of the study on patients’ symptoms and experience reporting. We aim to evaluate how using the app’s features correlates with patients’ reporting behaviors. Results from the heuristic evaluation suggest that more research is needed to underpin the cultural nuances of providing information about the disease considering patients’ social conventions.

## Abbreviations

FHIR: Fast Healthcare Interoperability Resources
NCR: National Cancer Registry
NCD: noncommunicable disease
OECD: Organization for Economic Co-operation and Development
PREM: patient-reported experience measure
PRO: patient-reported outcome
PROM: patient-reported outcome measure
